# Forsythiaside A Suppresses Ferroptosis and Mitigates Type 2 Diabetes Osteoporosis Through the NRF2/GPX4 Axis

**DOI:** 10.1002/fsn3.70991

**Published:** 2025-09-29

**Authors:** Yi‐xun Huang, Yi‐tian Bu, Ye‐kai Zhang, Yi‐kai Wang, Yang‐fan Guo, Chen Jin, Si‐xiang Feng, Lai‐fa Kong, Wei‐kai Chen, Lei Yang

**Affiliations:** ^1^ Department of Orthopaedics The Second Affiliated Hospital and Yuying Children's Hospital of Wenzhou Medical University Wenzhou Zhejiang China; ^2^ The Second School of Medicine Wenzhou Medical University Wenzhou Zhejiang China; ^3^ Zhejiang Provincial Key Laboratory of Orthopaedics Wenzhou Zhejiang China; ^4^ Emergency Department Affiliated Jinhua Hospital, Zhejiang University School of Medicine, Jinhua Municipal Central Hospital Jinhua China

**Keywords:** ferroptosis, forsythiaside A, GPX4, Nrf2, type 2 diabetes osteoporosis

## Abstract

Type 2 diabetes osteoporosis (T2DOP) is a chronic bone metabolic disorder that has led to substantial economic losses worldwide. Unlike conventional postmenopausal osteoporosis, the hyperglycemic microenvironment in T2DOP significantly heightens the risk of fractures and osteonecrosis. However, effective pharmacological interventions for T2DOP remain scarce. Research indicates that ferroptosis is crucial in the development of T2DOP. Forsythiaside A (FA), extracted from *Forsythia suspensa* fruit, demonstrates various biological activities such as anti‐inflammatory, antioxidant, neuroprotective effects, and ferroptosis inhibition. This study aims to investigate the effects and mechanisms of FA in the context of T2DOP. We developed T2DOP models both in vitro and in vivo and subsequently treated them with FA. The results demonstrated that FA effectively inhibited ferroptosis and mitigated T2DOP. Mechanistic studies indicate that FA may promote the nuclear translocation of nuclear factor erythroid 2‐related factor 2 (Nrf2), enhancing glutathione peroxidase 4 (GPX4) expression to inhibit ferroptosis. FA concurrently boosts the expression of proteins associated with osteogenesis. In conclusion, our study highlights FA as a potential therapeutic agent for the treatment of T2DOP.

## Graphical abstract

Type 2 diabetes‐associated osteoporosis (T2DOP), a metabolic bone disorder exacerbated by hyperglycemia‐induced ferroptosis, lacks effective therapies. Our study demonstrates that forsythiaside A (FA), a bioactive compound from *Forsythia suspensa* , alleviates T2DOP in preclinical models by dual mechanisms: activating the Nrf2/GPX4 axis to suppress ferroptosis and oxidative stress, and enhancing osteogenic protein expression to restore bone formation. These findings position FA as a novel multitarget therapeutic candidate for combating diabetic bone complications.

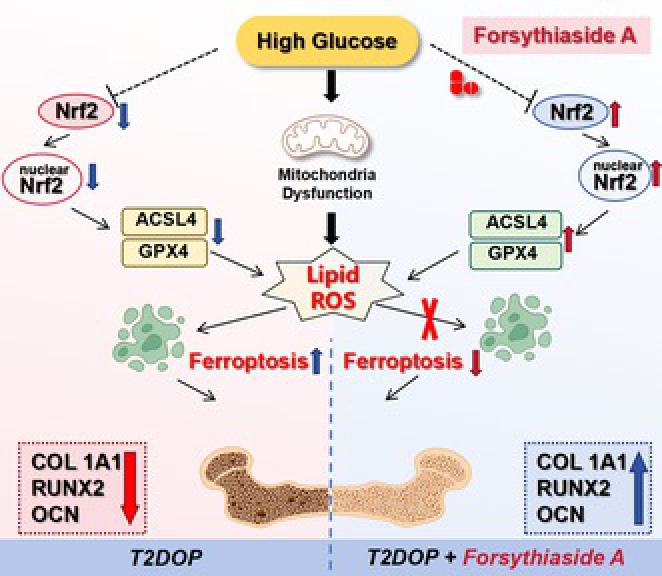

## 1 Introduction

The aging global population has led to a rising incidence of diabetes, resulting in a significant economic burden (Hu 2011; Zhang et al. 2010). The complications associated with diabetes, such as diabetic nephropathy and diabetic retinopathy, are the leading contributors to disability and mortality among patients (Cole and Florez 2020; Morrish et al. 2001). Type 2 diabetes (T2D) is the most common form of diabetes, comprising 90% of all cases (Chatterjee et al. 2017). Type 2 diabetic osteoporosis (T2DOP) is a metabolic bone disorder linked to type 2 diabetes, marked by impaired bone metabolism that results in significant structural abnormalities and decreased bone strength (Vestergaard 2007; Ma et al. 2012). Studies have demonstrated that, compared to nondiabetic individuals, those with diabetes have a 32% higher risk of fractures, presenting serious safety risks (Janghorbani et al. 2007; Hofbauer et al. 2007). Hence, there is an immediate requirement to delve into the underlying processes of T2DOP and identify possible therapeutic options. Ferroptosis, a crucial type of programmed cell death, was initially identified in 2012 (Jiang et al. 2021; Galluzzi et al. 2018). It is distinguished by higher intracellular iron levels, the buildup of reactive oxygen species (ROS), and the inactivation of the antioxidant enzyme GPX4 (Tang et al. 2021; Friedmann Angeli et al. 2014). Among them, GPX4 is an antioxidant enzyme negatively regulated by endoplasmic reticulum stress (Ingold et al. 2018). It converts lipid hydroperoxides into lipid alcohols, thereby suppressing the generation of Fe^2+^‐dependent ROS. Inhibition of GPX4 can induce ferroptosis (Carlson et al. 2016). Research indicates that diabetes can trigger ferroptosis in diverse cell types, contributing to the advancement of several diseases such as diabetic kidney disease, retinopathy, cardiomyopathy, and osteoporosis (Liu et al. 2024; Fernández‐Real et al. 2015; Feng et al. 2021; Baum et al. 2016). This imposes significant risks of disability and economic burdens on patients. Research indicates that ferroptosis in bone marrow mesenchymal stem cells (BMSCs) is a key mechanism in T2DOP (Tang et al. 2024; Jing et al. 2023). Therefore, inhibiting ferroptosis may rescue the death of BMSCs, thereby mitigating the progression of T2D‐related osteopathy.

Nuclear factor erythroid 2‐related factor 2 (Nrf2) is essential for regulating the antioxidant system, crucially combating oxidative stress and reducing mitochondrial damage (Chen 2022; Harder et al. 2015). Additionally, activation of Nrf2 has been shown to significantly inhibit ferroptosis, as validated in various cell types and disease contexts (Anandhan et al. 2023; Cui et al. 2021; Zhang, Li, et al. 2024). However, the relationship among Nrf2, ferroptosis, and T2DOP remains unexplored to date.

Forsythiside A (FA), the main active component extracted from Forsythia, is extensively utilized in clinical settings for its diverse pharmacological properties and notable biological effectiveness (Cao et al. 2022). Modern pharmacological studies have demonstrated that Forsythia exhibits various effects, including anti‐inflammatory, antimicrobial, antioxidant, and antipyretic properties (Wang et al. 2013). Studies have shown that FA can activate Nrf2, which in turn inhibits ferroptosis by upregulating the levels of GPX4 (Wang et al. 2022; Guo et al. 2024). No research has yet investigated the possible connection between ferroptosis and T2DOP.

We developed an in vitro T2DOP model by culturing BMSCs in a medium with 25.5 mM glucose to study ferroptosis's effects. For the in vivo model, rats were induced with T2DOP through high‐glucose and high‐fat (HGHF) feeding and streptozotocin (STZ) injection (Xu et al. 2024). The results indicated that FA can activate Nrf2 by promoting its nuclear translocation in BMSCs, thereby inhibiting ferroptosis in BMSCs under HGHF conditions. In animal models, FA significantly inhibited the initiation and development of T2DOP. Consequently, we have identified FA as a potential therapeutic agent for T2DOP.

## 2 Materials and Methods

### 2.1 Reagents and Antibodies

Forsythiaside A (CAS no. 79916‐77‐1, purity = 99.43%, C_29_H_36_O_15_) was purchased from MedChemExpress (Shanghai, China). Hoechst 33342, Reactive Oxygen Species Assay Kit, DAPI, and JC‐1 Mitochondrial Membrane Potential Assay Kit were procured from Beyotime (Hangzhou, China). DMEM/F12, fetal bovine serum (FBS), and phosphate‐buffered saline (PBS) were obtained from Gibco, Grand Island, NY, USA. Antibodies for ACSL4, GPX4, Opa1, and Drp1 were obtained from ABclonal (Wuhan, China). Mfn1 and Mfn2 were purchased from Cell Signaling Technology (Boston, USA). COL1A1, GAPDH, RUNX2, and OCN were purchased from Abcam (Cambridge, UK). Nrf2, Tom20, and Lamin B were purchased from Proteintech (Wuhan, USA).

### 2.2 BMSCs Culture

Six‐week‐old healthy Sprague–Dawley rats were euthanized via intraperitoneal injection of an overdose of 10% chloral hydrate. BMSCs were aseptically extracted from the femoral marrow cavity using PBS. The cell pellet was resuspended in DMEM/F12 with 15% FBS and 1% streptomycin/penicillin, then incubated at 37°C with 5% CO_2_ (Jin et al. 2022).

### 2.3 Ethynyl‐20‐Deoxyuridine Incorporation Assay

Cell proliferation of BMSCs was assessed using an EdU detection kit (Beyotime, Hangzhou, China). BMSCs were seeded at an appropriate density in 48‐well plates for EdU detection. Cells were treated with EdU for 4 h and then fixed with 4% formaldehyde for 30 min. The cell nucleus was stained with Hoechst dye following PBS washing. Fluorescent images were obtained via a fluorescence microscope, and ImageJ software (National Institutes of Health, Bethesda, MD, USA) was used to calculate the percentage of EdU‐positive cells (red fluorescence) compared to the total cell count (blue fluorescence). This percentage represents the proliferative capacity of BMSCs.

### 2.4 ROS Generation Determination

BMSCs were seeded at an appropriate density in 24‐well plates. Cells were pretreated as per experimental groups and incubated with dihydroethidium (DHE) in darkness at 37°C for 30 min. The cells were then rinsed with PBS and observed using a confocal microscope. Image analysis utilized Image Pro Plus software version 6.0.

### 2.5 Transmission Electron Microscopy

Transmission electron microscopy (TEM) was used to observe changes in mitochondrial morphology. Following various pretreatments, BMSCs were fixed in a 2.5% glutaraldehyde solution at 4°C for 16 h. The cells were then dehydrated, embedded, sectioned, and stained. Finally, cellular ultrastructure was visualized using a Hitachi H‐7650 transmission electron microscope.

### 2.6 Cell Proliferation Assay

BMSCs were cultured in a 96‐well plate at a suitable density and treated based on the specific experimental groups and objectives. The cells were then washed three times with PBS. The medium was substituted with one containing 10% CCK‐8 reagent (Beyotime, Hangzhou, China), followed by a 2‐h incubation at 37°C. The absorbance for each group was measured at 450 nm using a microplate reader.

### 2.7 FerroOrange and C11 BODIPY Staining

BMSCs were seeded at an appropriate density in confocal culture dishes and pretreated according to the experimental groups. Solutions of 1 μM FerroOrange (Dojindo, Japan) and 10 μM C11 BODIPY (Invitrogen, USA) were prepared and introduced to the confocal dishes for each group. Cells were maintained at 37°C for half an hour, followed by three washes with PBS, and fluorescence intensity was measured using a Zeiss laser scanning confocal microscope.

### 2.8 Immunofluorescence

BMSCs were seeded at an appropriate density in confocal culture dishes and subjected to group‐specific pretreatment protocols. The cells were treated with 4% paraformaldehyde for 15 min, rinsed with PBS, and made permeable using 0.1% Triton X‐100. Primary antibodies, diluted 1:100 in a 10% fetal bovine serum albumin solution, were incubated with the cells for 24 h. After PBS washes, cells were incubated in darkness with Alexa Fluor 488‐conjugated secondary antibodies (Jackson ImmunoResearch, West Grove, USA) for 2 h. After additional PBS washes, the cells were further incubated with DAPI for 5 min, washed again, and visualized using a confocal microscope. Images were collected and analyzed to evaluate the results.

### 2.9 Mitochondrial Membrane Potential Assay

The mitochondrial membrane potential of BMSCs was evaluated using the JC‐1 probe. BMSCs were seeded at the appropriate density in 24‐well plates and cultured for 24 h before receiving specific treatments for each group. Cells were incubated with JC‐1 dye at 37°C for 20 min, following the manufacturer's instructions, and subsequently washed three times with serum‐free DMEM. Cells were observed and imaged for analysis using fluorescence microscopy (Olympus, Tokyo, Japan).

### 2.10 Western Blot Analysis

Protein extracts were analyzed by electrophoresis using 12% SDS‐PAGE gels and electroblotted onto polyvinylidene difluoride membranes at 300 mA for 90 min. The membranes were blocked with 5% skimmed milk and then incubated with specific primary antibodies (ACSL4, GPX4, Opa1, Drp1, Mfn1, Mfn2, COL1A1, RUNX2, GAPDH, OCN, Nrf2, Tom20, and Lamin B) at a dilution of 1:1000 in a gentle rotary incubation at 4°C overnight. Horseradish peroxidase‐labeled secondary antibody was added and incubated with shaking for 1 h, and the color was developed by Enhanced Chemiluminescence Kit detection. Three samples were used in Western blot analysis for replicate experiments.

### 2.11 Osteogenic Induction and Detection of BMSCs

BMSCs were appropriately seeded in 24‐well culture plates. Cells at 70%–80% confluence were differentiated into osteoblasts using the Osteogenic Differentiation Kit (OriCell, Guangzhou, China). Following a week of differentiation, cells underwent two PBS washes and were fixed with 4% paraformaldehyde for 15 min. Alkaline phosphatase (ALP) activity was evaluated using the BCIP/NBT color development kit (Beyotime, Hangzhou, China) according to the manufacturer's guidelines. After 21 days of differentiation, the cells underwent Alizarin Red S (ARS) staining. Finally, images were captured using an optical microscope (Nikon Eclipse 80i, Japan).

### 2.12 Malondialdehyde (MDA) and Glutathione (GSH) Measurement

BMSCs from each group were subjected to their specific treatments and then lysed at 4°C for 30 min. Following the manufacturer's instructions, lipid peroxidation (MDA) levels and reduced glutathione (GSH) content were measured and quantified using the respective assay kits.

### 2.13 Molecular Docking

Molecular docking studies were conducted for FA and Nrf2/GPX4 based on established protocols. The structural formula of FA was obtained from the PubChem database (https://pubchem.ncbi.nlm.nih.gov/). The three‐dimensional structures of Nrf2 and GPX4 were retrieved from the Protein Data Bank (https://www.rcsb.org/). Before docking, ligand and protein structures were preprocessed with AutoDock software (http://vina.scripps.edu/). The preprocessing comprised dehydration, hydrogenation, amino acid modification, energy optimization, and force field parameter adjustment, leading to molecular docking. Binding affinity (kcal/mol) was used to assess ligand–protein interaction stability, where higher values denote stronger interaction forces. Three‐dimensional docking images were visualized using UCSF PyMOL software, while two‐dimensional interaction diagrams were generated with LigPlus software.

### 2.14 Mitochondrial Function Assays

MitoSOX dye was used to assess mitochondrial ROS in BMSCs. BMSCs were initially seeded at a suitable density in a 24‐well plate and cultured for 24 h before undergoing the necessary pretreatment as per experimental protocols. Cells were incubated with MitoSOX dye at 37°C for 30 min as per the manufacturer's instructions, followed by three washes with serum‐free DMEM. Fluorescence microscopy (Olympus, Tokyo, Japan) was employed to observe the cells and capture images for further analysis.

### 2.15 Animal Models Play a Crucial Role in Evaluating the Efficacy of Drug Therapies

Eight‐week‐old Sprague–Dawley rats were randomly assigned to five groups, each containing five rats: Sham, T2DOP, T2DOP + FA (80 mg/kg), and T2DOP + FA (80 mg/kg) + ML385. The T2DOP rat model was developed using an 8‐week high‐sugar, high‐fat diet combined with low‐dose streptozotocin (STZ) injections. FA or ML385 (an Nrf2 inhibitor) was administered to T2DOP model rats at varying concentrations. The rats were housed in clean, well‐ventilated rooms with temperatures maintained at 24°C–26°C, and they had unlimited access to food and water. After 8 weeks of treatment, all animals were humanely euthanized for further study.

### 2.16 Microcomputerized Tomography (Micro‐CT) Analysis

Micro‐CT imaging of the rat distal femur was conducted at 70 kV and 200 μA, achieving a spatial resolution of 14.8 μm, with images reconstructed from 3D data. The volume of interest (VOI) for trabecular bone microarchitecture analysis comprised 100 CT slices, spanning from 2 mm below the growth plate to the distal end. The Micro‐CT system's built‐in software enabled the analysis of essential parameters such as trabecular bone volume fraction (BV/TV), trabecular thickness (Tb.Th, mm), trabecular number (Tb.N, 1/mm), and trabecular separation (Tb.Sp, mm).

### 2.17 Histology and Immunohistochemistry

After pretreatment, rats were euthanized, and their femora were collected and fixed in 4% paraformaldehyde for 24 h. The femora were decalcified in 10% EDTA for 28 days, cleared with xylene, and embedded in paraffin. Tissue sections, 4 μm thick, were prepared and stained using standard protocols with hematoxylin and eosin (H&E) and Masson's trichrome. The sections were incubated with primary antibodies targeting OPN, BMP2, Nrf2, and GPX4, followed by immunofluorescence and immunohistochemical staining. Immunoreactivity was evaluated following the manufacturer's guidelines using a horseradish peroxidase detection system (Vector Laboratories, Burlingame, CA, USA). Quantitative analysis was performed using ImageJ software.

### 2.18 Calcein‐Alizarin Red S Double Labeling

On the day following the procedure and prior to euthanasia, rats were administered intraperitoneal injections of calcein green and ARS at a dose of 30 mg/kg, both purchased from Sigma (Darmstadt, Germany). Post‐euthanasia, the bones were fixed, dehydrated, and embedded in Embed‐812 from Electron Microscopy Sciences (Hatfield, PA, USA). The bone specimens were sectioned into 5‐μm‐thick slices using the RM2265 hard tissue microtome (Leica, Wetzlar, Germany). Sections were visually examined and recorded using an Olympus Life Science microscope (Tokyo, Japan).

### 2.19 Statistical Analysis

The experiments were conducted three times to ensure consistency and reproducibility. Data are expressed as the mean ± standard deviation. GraphPad Prism 5.0 software was used for statistical analyses. A Student's *t*‐test or one‐way ANOVA was used. Statistical significance was determined with a *p* value threshold of 0.05 (*p* < 0.05).

## 3 Results

### 3.1 HGHF Can Induce Ferroptosis in BMSCs

**FIGURE 1:**
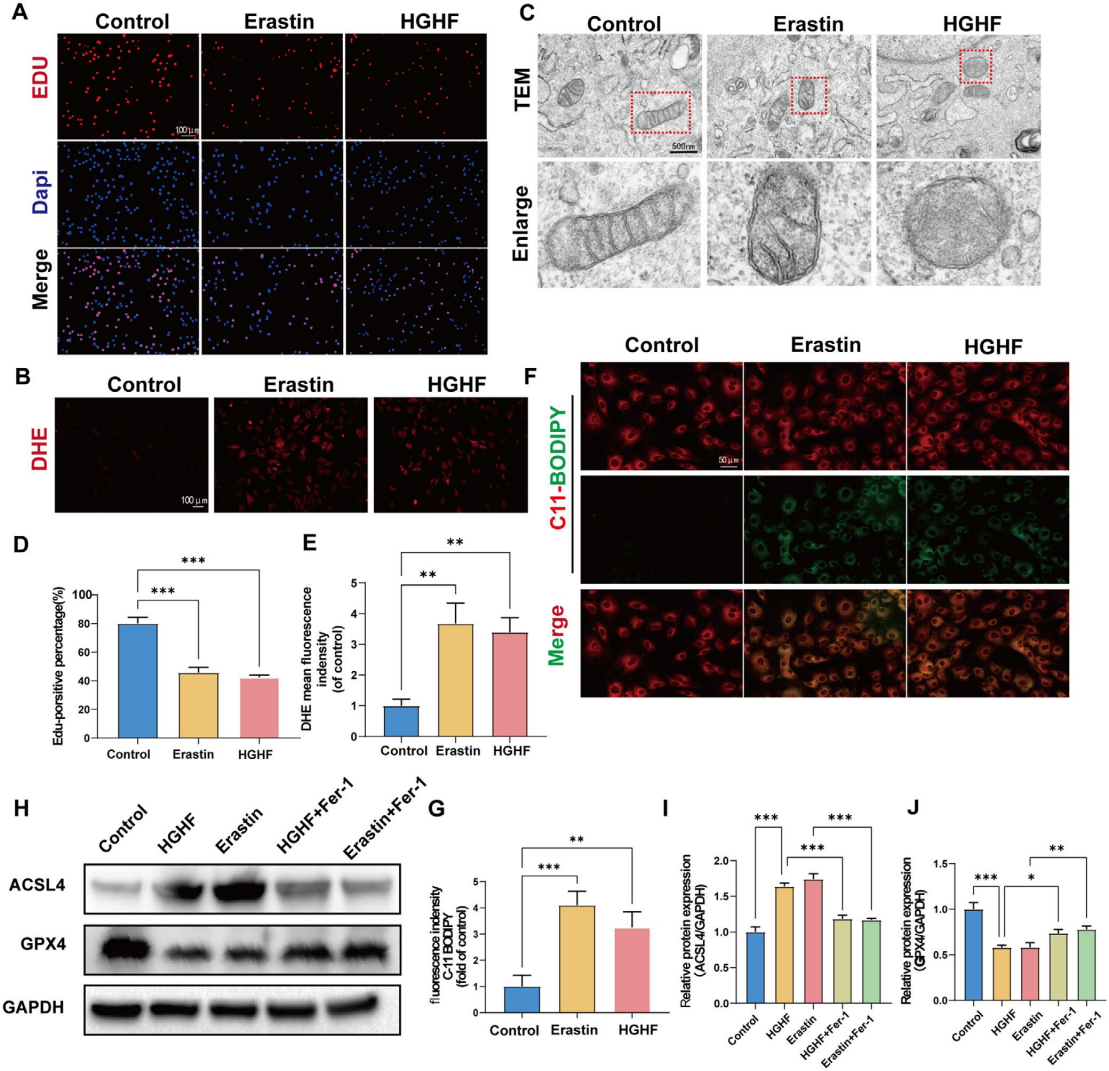
(A, D) HGHF triggers ferroptosis in BMSCs. Representative fluorescent images and quantitative analysis of BMSCs labeled with EdU (red) are presented, with nuclei visualized using Hochest 3342 staining (blue). (B, E) Representative fluorescent images and quantification analysis of BMSCs ROS levels with DHE staining. (C) Exemplary images depicting mitochondrial morphology in BMSCs subjected to various treatments. (F, G) Representative fluorescent images and quantification analysis of C11‐BODIPY staining in differentially treated BMSCs. Western blot analysis (H, I‐ J) was conducted to evaluate GPX4 and ACSL4 expression in BMSCs subjected to various treatments. The figures display the mean values ± SEM from three independent experiments, each performed in duplicate. Significance levels are indicated as follows: *p < 0.05, **p < 0.01, ***p < 0.001, and ****p < 0.0001. BMSCs, bone marrow mesenchymal stem cells; DHE, dihydroethidium; EdU, 5‐ethynyl‐2′‐deoxyuridine; GPX4, glutathione peroxidase 4; ROS, reactive oxygen species; WB, Western blot.

Studies have demonstrated that ferroptosis is a pivotal mechanism driving the death of BMSCs and promoting the progression of T2DOP. Furthermore, HGHF culture conditions have been shown to induce ferroptosis in BMSCs. To verify the ability of HGHF to induce ferroptosis, BMSCs were categorized into three groups: Control, Erastin, and HGHF. These groups were cultured in media supplemented with 5.5 mM glucose, 1 μM erastin (a ferroptosis inducer), and 25.5 mM glucose, respectively. EdU staining revealed that HGHF and erastin can suppress BMSCs proliferation (Figure 1A,D). DHE staining demonstrated that HGHF and erastin treatment elevated both the count of DHE‐positive cells and the DHE fluorescence intensity (Figure 1B,E). This suggests that HGHF treatment can increase ROS production in BMSCs. Mitochondrial TEM revealed that BMSCs in the Erastin and HGHF groups exhibited smaller mitochondria with damaged membranes (Figure 1C). Subsequently, C11 BODIPY was employed to assess lipid peroxidation levels in BMSCs across different groups. The results indicated that both erastin and HGHF treatment elevated lipid peroxidation levels in BMSCs (Figure 1F,G). The above findings demonstrate that under HGHF treatment, BMSCs exhibit proliferation inhibition, increased ROS production, and mitochondrial membrane damage. To verify that the observed phenomena were caused by HGHF‐induced ferroptosis, additional groups were included: the Erastin + Fer‐1 group and the HGHF + Fer‐1 group. Among them, Fer‐1, a ferroptosis inhibitor, was used at a concentration of 10 μM. Western blot revealed that both erastin and HGHF significantly upregulated the ferroptosis‐promoting protein ACSL4 and notably downregulated the ferroptosis‐protective protein GPX4. Furthermore, Fer‐1 effectively counteracted the changes in ACSL4 and GPX4 protein expression induced by erastin and HGHF (Figure 1H–J). Taken together, we conclude that HGHF can induce ferroptosis in BMSCs.

### 3.2 FA Can Inhibit HGHF‐Induced Apoptosis in BMSCs

**FIGURE 2:**
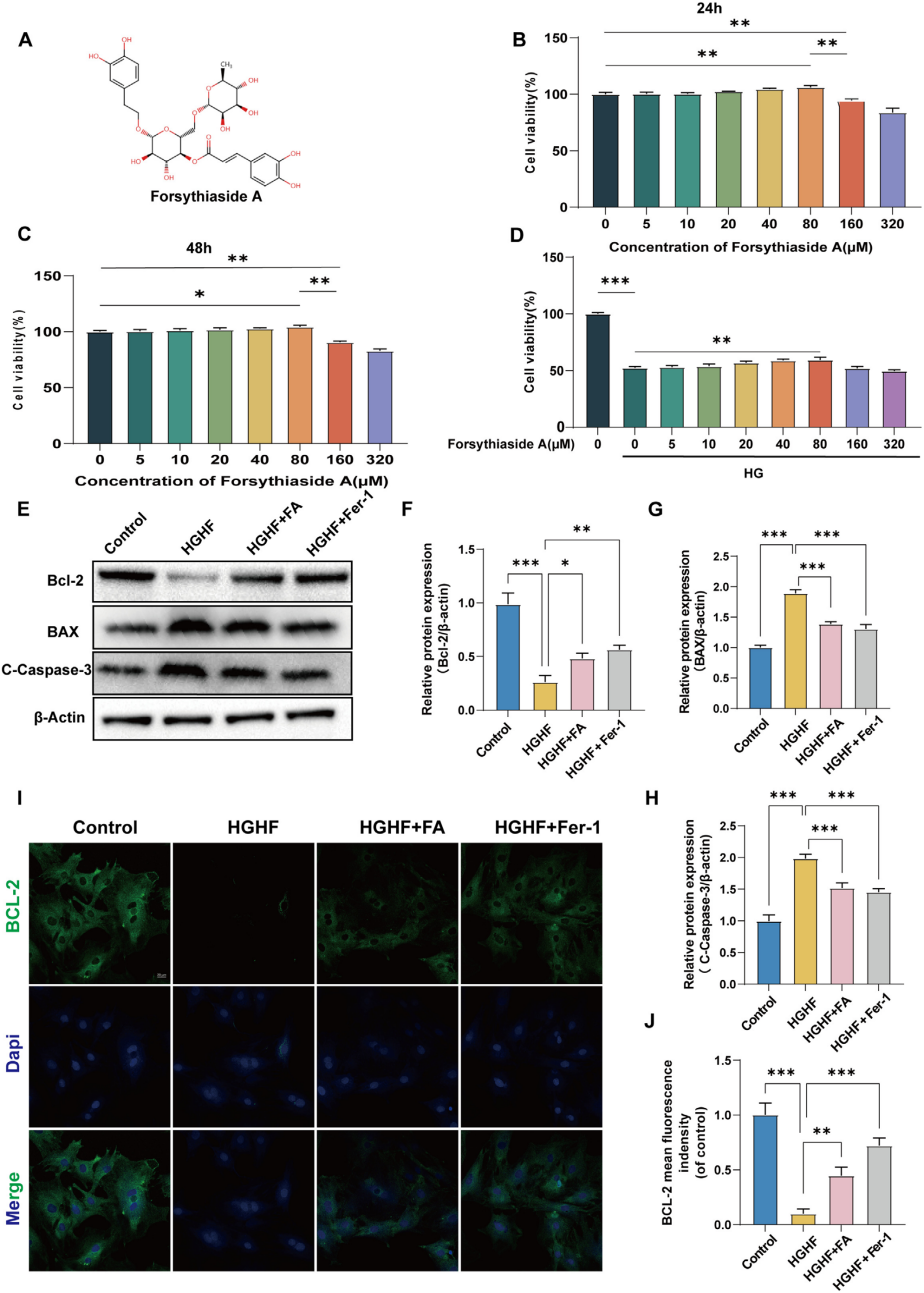
FA attenuates HGHF‐induced BMSCs apoptosis. (A) Chemical structure of FA. (B, C) Percentage of surviving cells after treatment with different concentrations of FA for 24 and 48 h. (D) Percentage of viable cells treated with HGHF in the presence or absence of varying FA concentrations. Expression levels of Bax, Bcl‐2, and cleaved Caspase‐3 in BMSCs were analyzed across various treatment groups.(E‐H) Expression levels of Bax, Bcl‐2 and Cleaved Caspase‐3 in BMSCs of each group after different treatments. (I, J) and quantification of Bcl‐2 fluorescence, an antiapoptosis‐related protein, are presented for each group. Graph data represent means ± SEM from triplicate experiments. *p < 0.05, **p < 0.01, ***p < 0.001, and ****p < 0.0001.

The chemical structure of ferulic acid (FA) is presented in Figure 2A. To establish the optimal therapeutic window for FA application, we conducted a systematic concentration gradient screening (5–320 μM) using CCK‐8 cytotoxicity assays on BMSCs. As shown in Figure 2B,C, 24/48‐h exposure to 160 μM FA caused a significant cell viability reduction (*p* < 0.01 vs. control), whereas concentrations ≤ 80 μM showed no detectable cytotoxicity. Based on this safety profile, we selected the 5–80 μM range for subsequent ferroptosis protection studies. Notably, among the tested concentrations, 80 μM FA demonstrated striking efficacy in counteracting high glucose/high fat (HGHF)‐induced ferroptosis, as evidenced by restored cell viability markers (Figure 2D). Given the critical role of apoptosis in cellular homeostasis, we examined BCL‐2 protein family dynamics. Western blot analysis revealed that HGHF exposure significantly downregulated antiapoptotic BCL‐2 expression while upregulating proapoptotic Bax and cleaved caspase‐3 (C‐Casp3) levels (Figure 2E–H). Importantly, FA treatment reversed these apoptotic markers to an extent comparable with the ferroptosis inhibitor Ferrostatin‐1 (Fer‐1). Immunofluorescence analysis corroborated these findings, demonstrating enhanced BCL‐2 signal intensity and reduced C‐Casp3 activation in FA‐treated groups compared to HGHF controls (Figure 2I,J). These multimodal observations collectively indicate that FA protects BMSCs against HGHF‐induced damage through dual modulation of ferroptosis and apoptosis pathways.

### 3.3 FA Attenuates Ferroptosis Caused by HGHF in BMSCs

**FIGURE 3:**
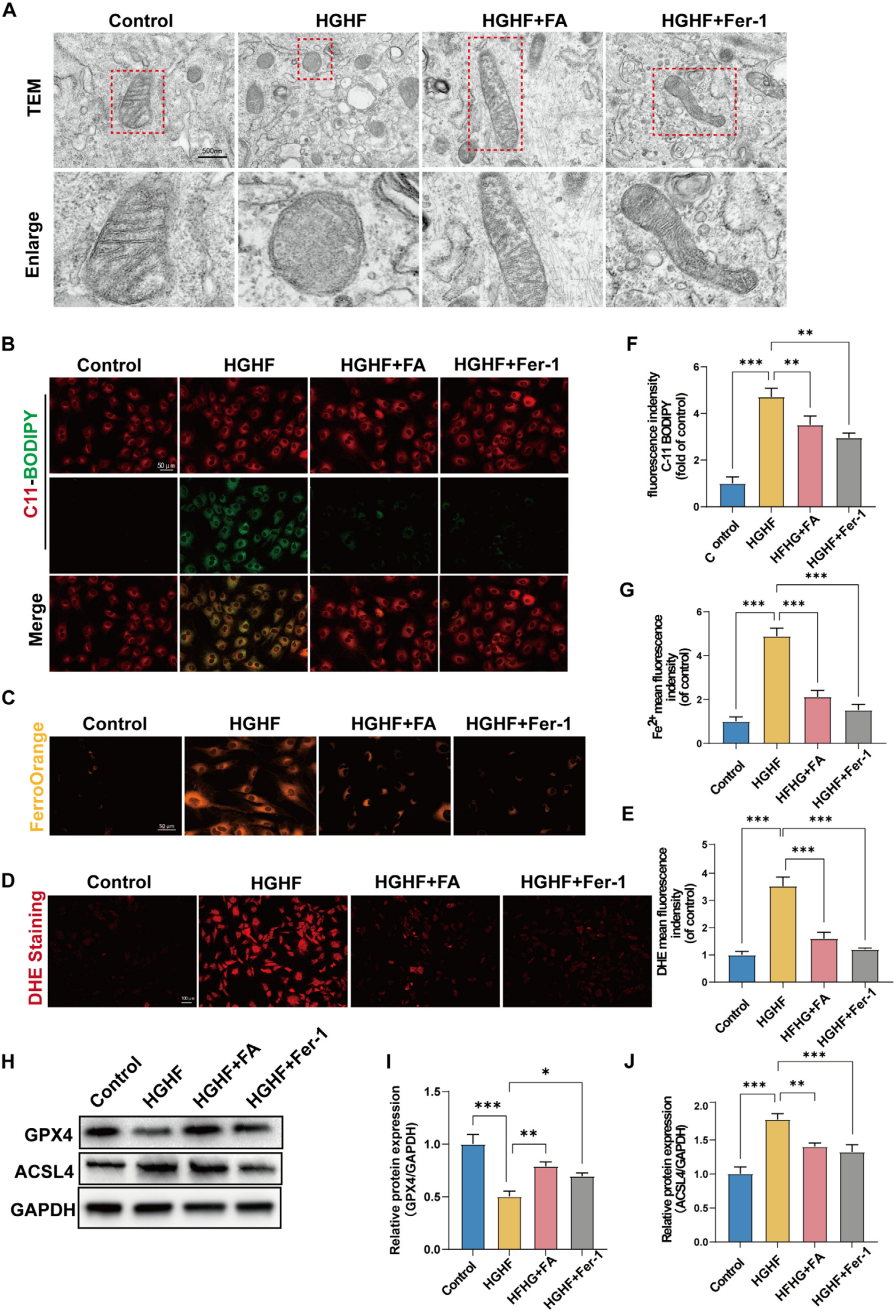
FA mitigates HGHF‐triggered ferroptosis in BMSCs. (A) Representative images depicting the mitochondrial morphology of BMSCs under various treatment conditions. (B, F) Representative fluorescence images and corresponding quantitative plots of C11‐BODIPY staining in BMSCs treated under different conditions. (C, G) Representative fluorescence images and quantitative analysis of FerroOrange staining in BMSCs were conducted under different treatment conditions. (D, E) Quantification of ROS levels in BMSCs treated under different conditions, assessed by DHE staining. (H–J) WB analysis of GPX4 and ACSL4 expression in different treated BMSCs. GPX4, glutathione peroxidase 4; ROS, reactive oxygen species; WB, Western blot. *p < 0.05, **p < 0.01, ***p < 0.001, and ****p < 0.0001.

We subsequently investigated whether FA could inhibit HGHF‐induced ferroptosis in BMSCs. The cells were categorized into four groups: Control, HGHF, HGHF + FA, and HGHF + Fer‐1, each subjected to its respective treatment. TEM imaging of the cells revealed that mitochondria in the HGHF group exhibited swollen and disrupted cristae, while the mitochondria in the HGHF + FA and HGHF + Fer‐1 groups maintained intact cristae (Figure 3A). Additionally, C11‐BODIPY staining was performed to assess lipid peroxidation levels. The heightened green fluorescence intensity signified increased lipid peroxidation, with staining results revealing elevated lipid peroxidation in HGHF‐treated BMSCs. However, treatment with FA and Fer‐1 markedly decreased lipid peroxidation levels (Figure 3B,F). FerroOrange serves as a probe for Fe^2+^ ions. Cells from various groups were stained with FerroOrange, revealing a notable rise in fluorescence intensity after HGHF treatment. In contrast, FA and Fer‐1 treatments led to a reduction in fluorescence intensity (Figure 3C,G). ROS levels were evaluated with the DHE fluorescence probe, revealing that FA and Fer‐1 reduced ROS levels in HGHF‐treated BMSCs (Figure 3D,E). Western blot analysis indicated that HGHF treatment increased ACSL4 expression, a protein that promotes ferroptosis, while decreasing GPX4 expression, a protein that protects against ferroptosis. Treatment with FA and Fer‐1 inhibited these changes in protein expression (Figure 3H–J). Collectively, these data suggest that FA can suppress HGHF‐induced ferroptosis in BMSCs.

### 3.4 FA Mitigates the Mitochondrial Dysfunction in HGHF‐Induced BMSCs

**FIGURE 4:**
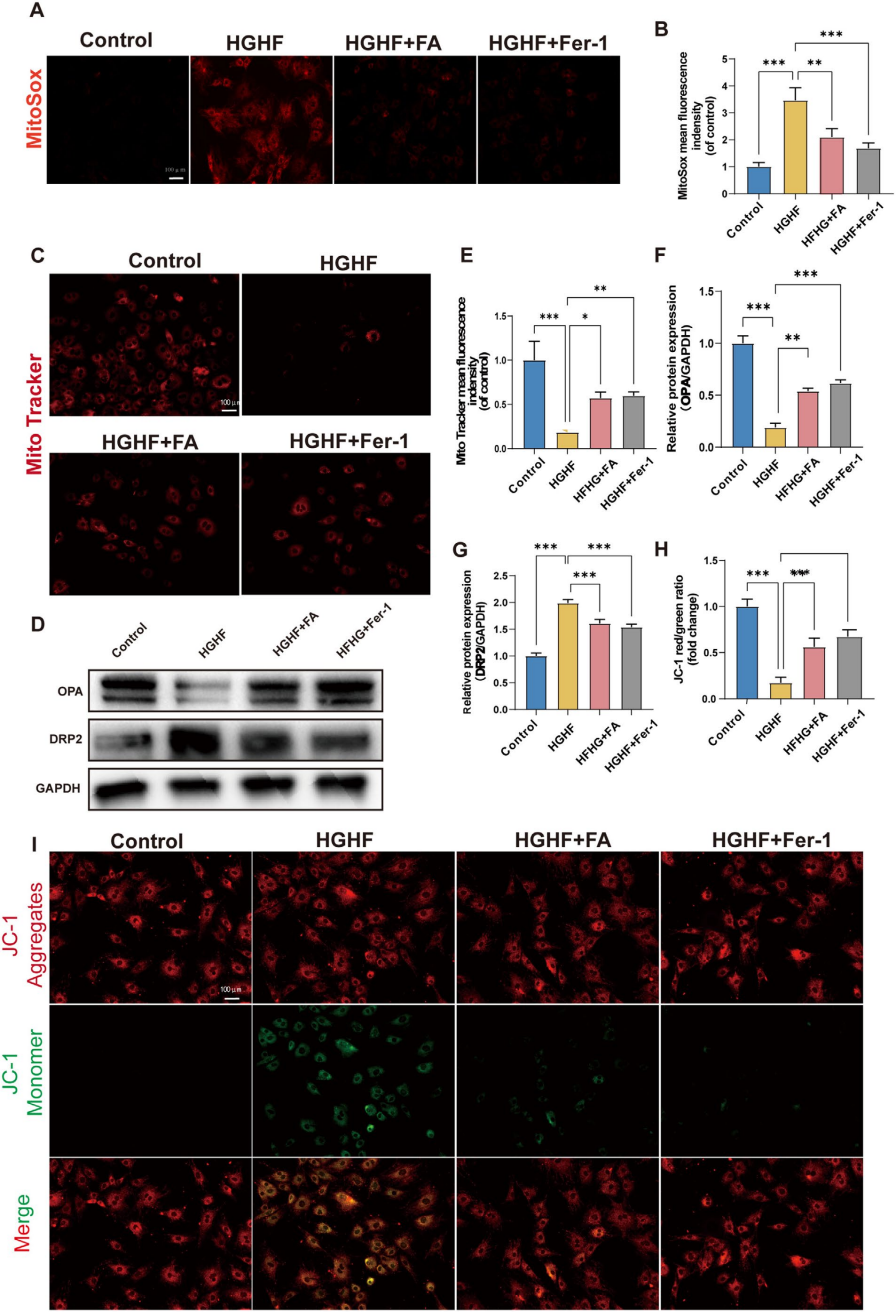
FA reverses mitochondrial dysfunction HGHF‐induced in BMSCs. (A, B) Mitochondrial reactive oxygen species (mtROS) staining was performed using MitoSox (red). The paper presents representative images and quantitative analysis of MitoSox staining in red. (C, E) Representative images and quantification of MitoTracker (red) staining. (D, G, H) Western blot analysis was conducted to assess OPA1 and DRP1 expression in BMSCs subjected to various treatments. (F, I) Confocal fluorescence microscopy detected JC‐1 aggregates (red) and monomers (green) in BMSCs. The figures display the mean values ± SEM from three independent experiments, each performed in duplicate. Abbreviations used include mtROS for mitochondrial reactive oxygen species and WB for Western blot. *p < 0.05, **p < 0.01, ***p < 0.001, ****p < 0.0001.

A hallmark of ferroptosis is the alteration of mitochondrial structure and the impairment of mitochondrial function. To assess mitochondrial ROS (mtROS), we used the MitoSox probe. The results showed a significant increase in fluorescence intensity following HGHF treatment, indicating elevated mtROS levels. However, treatment with FA and Fer‐1 significantly reduced mtROS accumulation, suggesting that FA can suppress oxidative stress induced by HGHF in BMSCs (Figure 4A,B). MitoTracker and JC‐1 probes were utilized to assess mitochondrial membrane potential (MMP). Results from MitoTracker staining indicated that HGHF treatment reduced the fluorescence intensity in BMSCs, whereas treatment with FA and Fer‐1 successfully restored the fluorescence intensity (Figure 4C,E). JC‐1 staining indicated that HGHF treatment elevated green fluorescence intensity, suggesting oxidative stress, whereas FA and Fer‐1 treatments effectively reduced this intensity (Figure 4F,I). Western blot analysis indicated that HGHF treatment reduced OPA1 expression, which is crucial for mitochondrial inner membrane fusion, and elevated DRP1 expression, a protein associated with mitochondrial fission. In contrast, treatment with FA and Fer‐1 increased OPA1 expression and reduced DRP1 expression (Figure 4D,G,H). Collectively, these results suggest that FA alleviates mitochondrial dysfunction induced by HGHF.

### 3.5 FA Counteracts the Suppression of Osteogenic Differentiation and Mineralization Caused by HGHF in BMSCs

**FIGURE 5:**
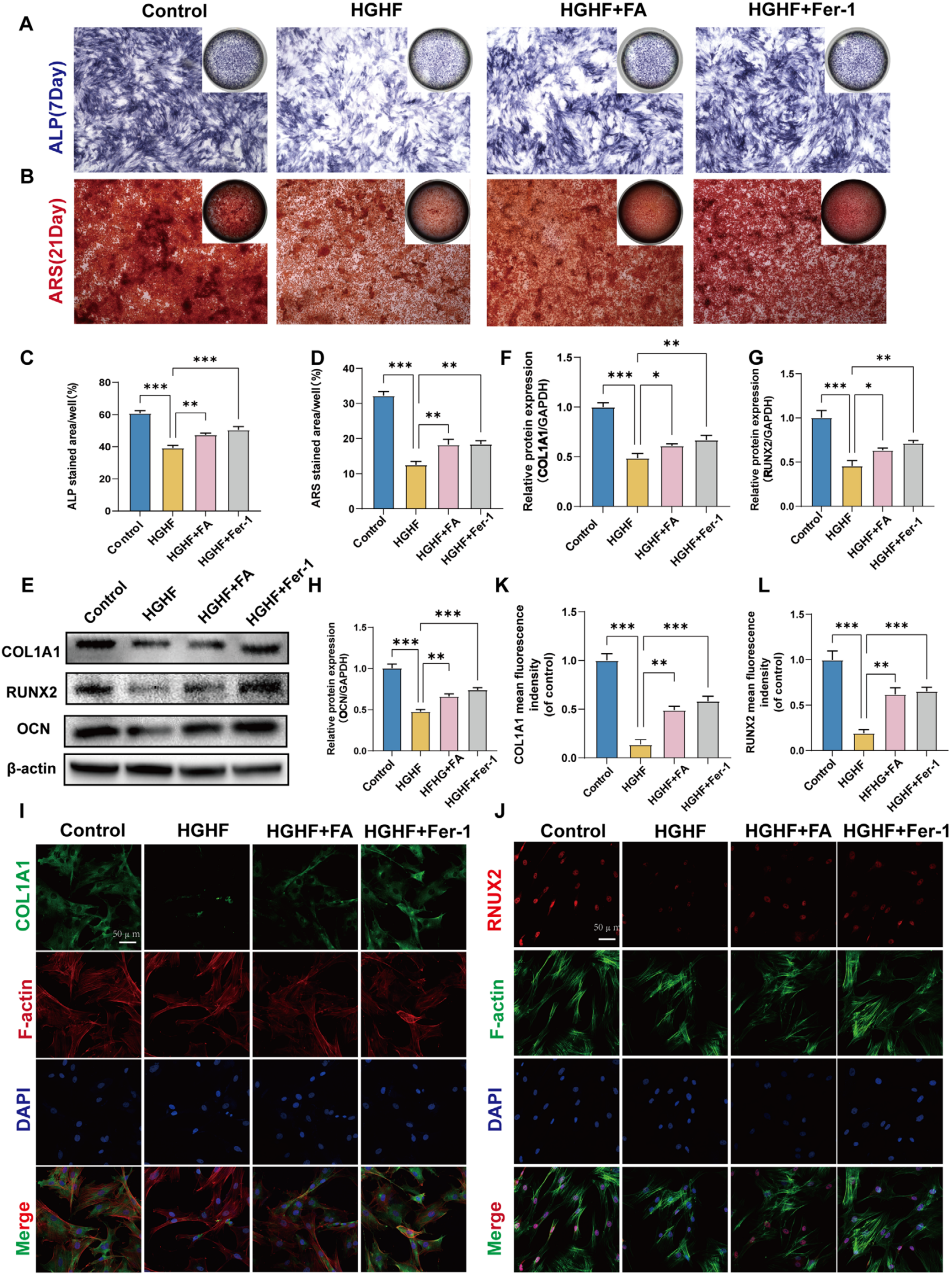
FA enhances BMSCs function by inhibiting ferroptosis. (A–D) Representative images and quantitative analysis of ALP staining on Day 7 and ARS staining on Day 21 during osteogenic induction of variably treated BMSCs. (E–H) Western blot analysis of COL1A1, OCN, and RUNX2 expression in BMSCs under various treatments. (I–L) Representative images and quantification of osteogenesis‐related proteins COL1A1 and RUNX2 fluorescence for each group following various treatments. The figures display the mean values ± SEM from three independent experiments, each performed in duplicate. ALP, alkaline phosphatase; ARS, Alizarin Red S; WB, Western blot. *p < 0.05, **p < 0.01, ***p < 0.001, ****p < 0.0001.

Research indicates that FA can prevent HGHF‐induced ferroptosis in BMSCs. We examined if FA could counteract the suppression of osteogenic differentiation and mineralization in BMSCs caused by HGHF treatment. Alkaline phosphatase (ALP) and Alizarin Red S staining demonstrated that HGHF treatment suppressed calcium nodule formation. However, treatments with FA and Fer‐1 counteracted the HGHF‐induced inhibition of osteogenic differentiation and mineralization in BMSCs (Figure 5A–D). Moreover, western blot revealed that the expression of specific markers, including COL1A1, RUNX2, and OCN, was reduced following HGHF treatment. Treatment with FA significantly elevated the expression of these proteins, reinforcing our findings (Figure 5E–H). Additionally, immunofluorescence staining of COL1A1 and RUNX2 corroborated the western blot findings (Figure 5I–L). Collectively, these results suggest that FA can inhibit HGHF‐induced osteogenic differentiation and mineralization in BMSCs.

### 3.6 FA Inhibits Ferroptosis in BMSCs Through Activating Nrf2

**FIGURE 6:**
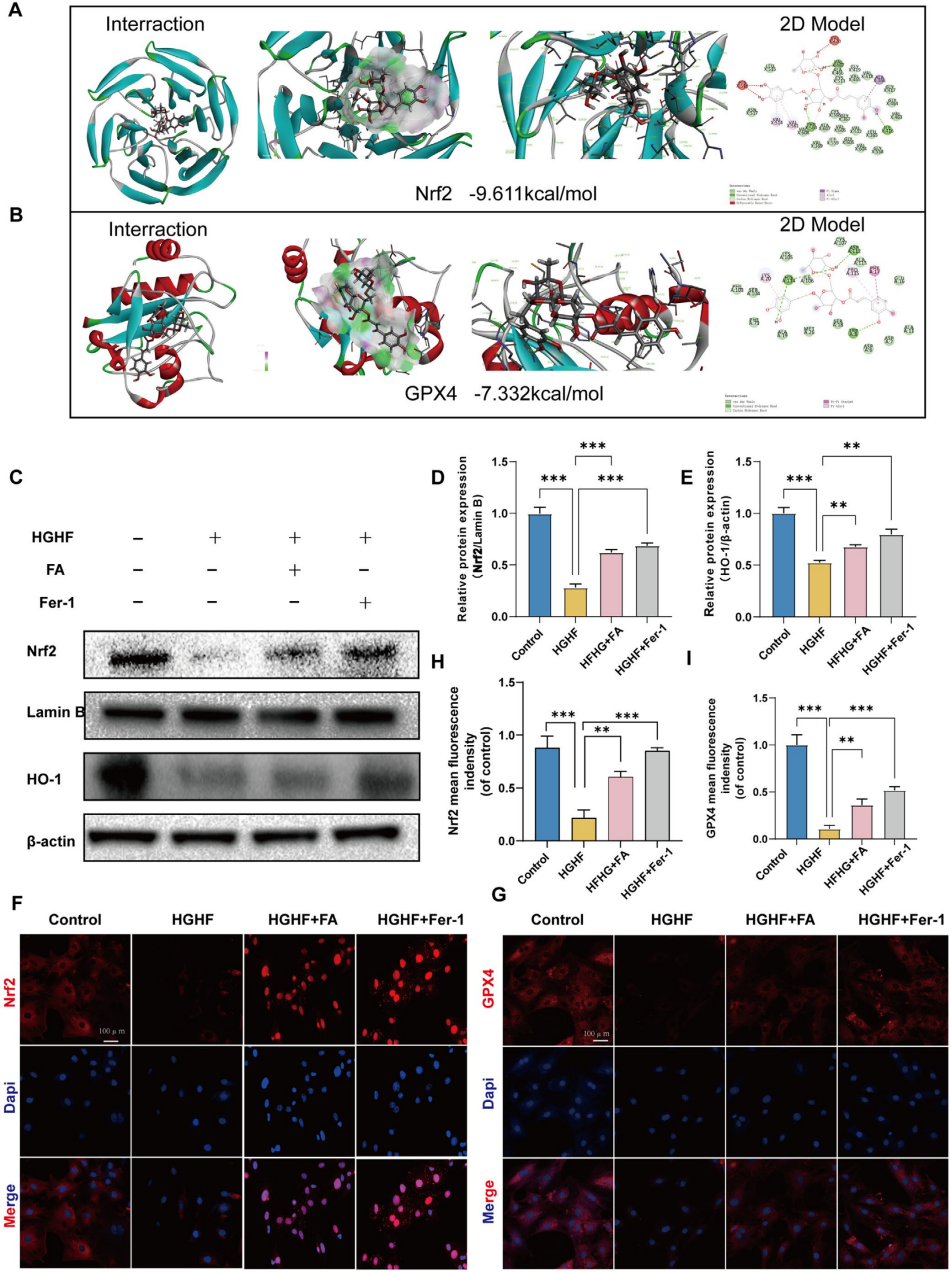
FA suppresses HGHF‐induced ferroptosis in BMSCs through the Nrf2/GPX4 pathway. (A, B) A ribbon model is used to represent protein residues, illustrating the 3D binding model. The binding site affinities of −9.611 and −7.332 kcal/mol were observed for Fra docking with Nrf2 and GPX4, respectively. A space‐filling model illustrates FA binding within the Nrf2 and GPX4 pockets. (C–E) Western blot analysis was conducted to assess Nrf2 and HO‐1 expression in BMSCs under various treatment conditions. The figures display the mean values ± SEM from three independent experiments, each performed in duplicate. (F, G) Immunofluorescence analysis of Nrf2 and GPX4 in BMSCs subjected to various treatments. **p < 0.01, ***p < 0.001, ****p < 0.0001.

Nrf2 is crucial for cellular defense against oxidative stress, essential for maintaining redox balance. GPX4, on the other hand, is a critical protein that inhibits ferroptosis. We hypothesized that FA suppresses HGHF‐induced ferroptosis in BMSCs through the Nrf2/GPX4 pathway. To test this, we first conducted molecular docking simulations of FA with Nrf2 or GPX4, and the results showed that FA binds and attaches to the docking sites of both Nrf2 and GPX4. The binding energies of hydrogen bonding interactions between FA and Nrf2 or GPX4 were 9.611 and 7.332 kcal/mol, respectively (Figure 6A,B). Western blot analysis showed that HGHF treatment significantly reduced Nrf2 protein expression, whereas FA and Fer‐1 treatments restored it (Figure 6C–E). Immunofluorescence analysis demonstrated that FA and Fer‐1 facilitated Nrf2 nuclear translocation and prevented HGHF‐induced GPX4 degradation (Figure 6F,G). In conclusion, our experiments provide evidence that FA can bind to Nrf2 and GPX4, promote the nuclear translocation of Nrf2, and increase the expression of GPX4, thereby inhibiting HGHF‐induced ferroptosis in BMSCs.

### 3.7 FA Treatment Restored Bone Microstructure In Vivo

**FIGURE 7:**
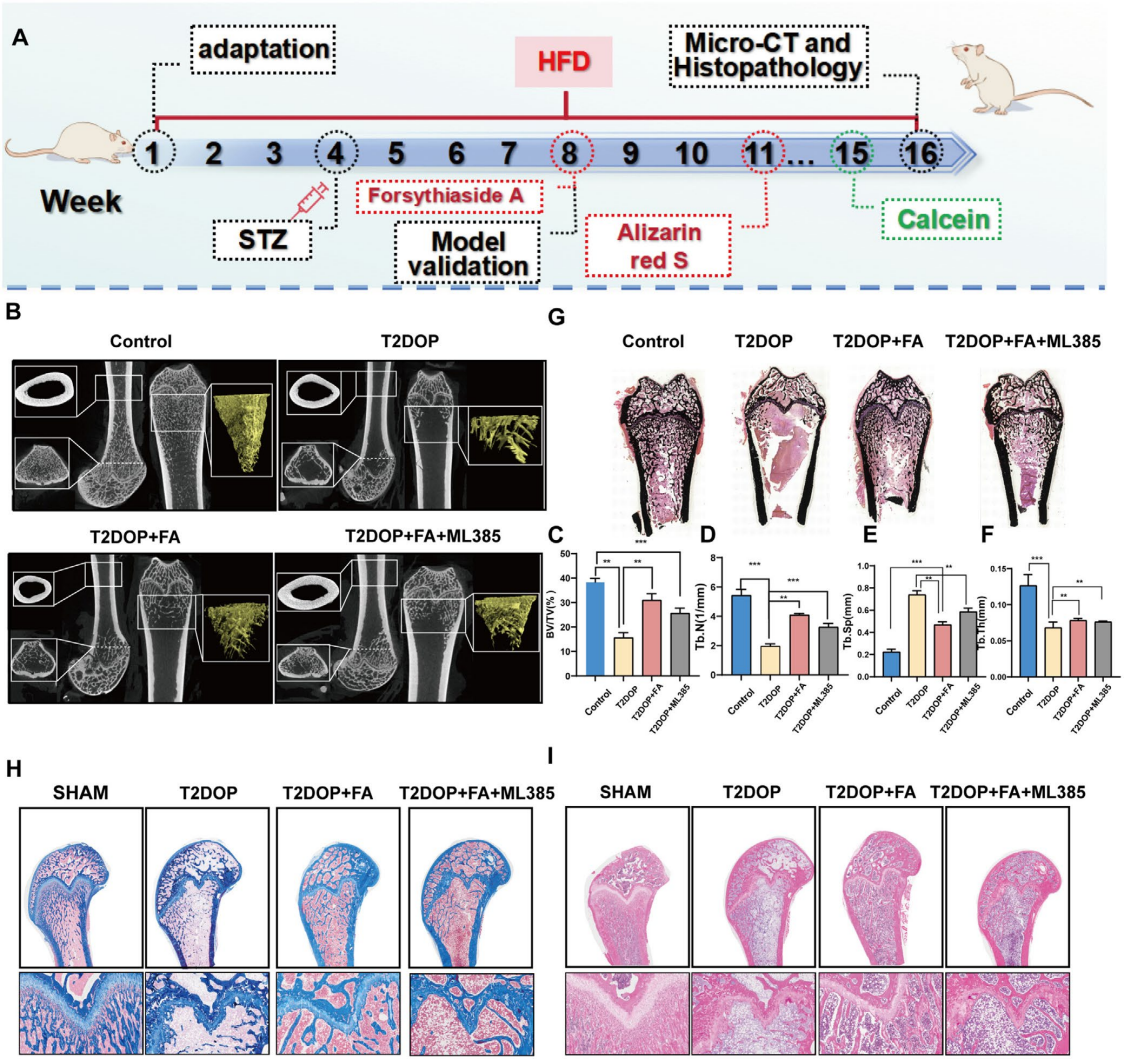
FA treatment revived the microstructure of the T2DOP rat. (A) A schematic illustrates the timeline for T2DOP establishment, parameter collection, and FA introduction in the animal study. (B–F) Representative micro‐CT images display longitudinal and transverse sections of distal femurs, along with BV/TV, Tb.N, Tb.Sp, and Tb.Th metrics for animals under varied treatments. (G) VonKossa staining of the distal end of the femur in different groups. (H, I) Representative images of metaphyseal tissue sections from the thigh using H&E and Masson's staining. The figures display the mean values ± SEM from three independent experiments, each performed in duplicate. BV/TV represents trabecular bone volume, while micro‐CT stands for microcomputerized tomography. Tb.N, Tb.Sp, and Tb.Th denote trabecular number, trabecular separation, and trabecular thickness, respectively.

Studies have demonstrated that FA can inhibit ferroptosis in BMSCs through the activation of Nrf2. We developed a T2DOP rat model by combining a high‐fat, high‐glucose diet with low‐dose streptozotocin (STZ) injections to examine the in vivo effects of FA (Figure 7A). Micro‐CT imaging 3D reconstruction demonstrated significant trabecular bone structure reduction at the distal femur following 8 weeks of a high‐fat, high‐glucose diet combined with low‐dose STZ injection. However, treatment with FA for an additional 8 weeks significantly ameliorated the trabecular bone structure at the femoral distal end. Quantitative data demonstrated that FA treatment significantly enhanced the bone volume to total volume ratio (BV/TV), trabecular number (Tb.N), and trabecular thickness (Tb.Th), while notably reducing trabecular separation (Tb.Sp). ML385, a specific inhibitor of Nrf2, was employed, and results showed that coadministration of FA and ML385 attenuated the therapeutic effect of FA (Figure 7B–F). Histological examination with HE and Masson's trichrome staining indicated a rise in trabeculae count in the FA‐treated group (Figure 7H,I). Von Kossa staining showed consistent results concerning bone morphological modifications (Figure 7G). Collectively, these findings suggest that FA effectively inhibits the progression of T2DOP in vivo.

### 3.8 FA Treatment In Vivo Reinstated the Expression of Nrf2 and Proteins Associated With Osteogenesis

**FIGURE 8:**
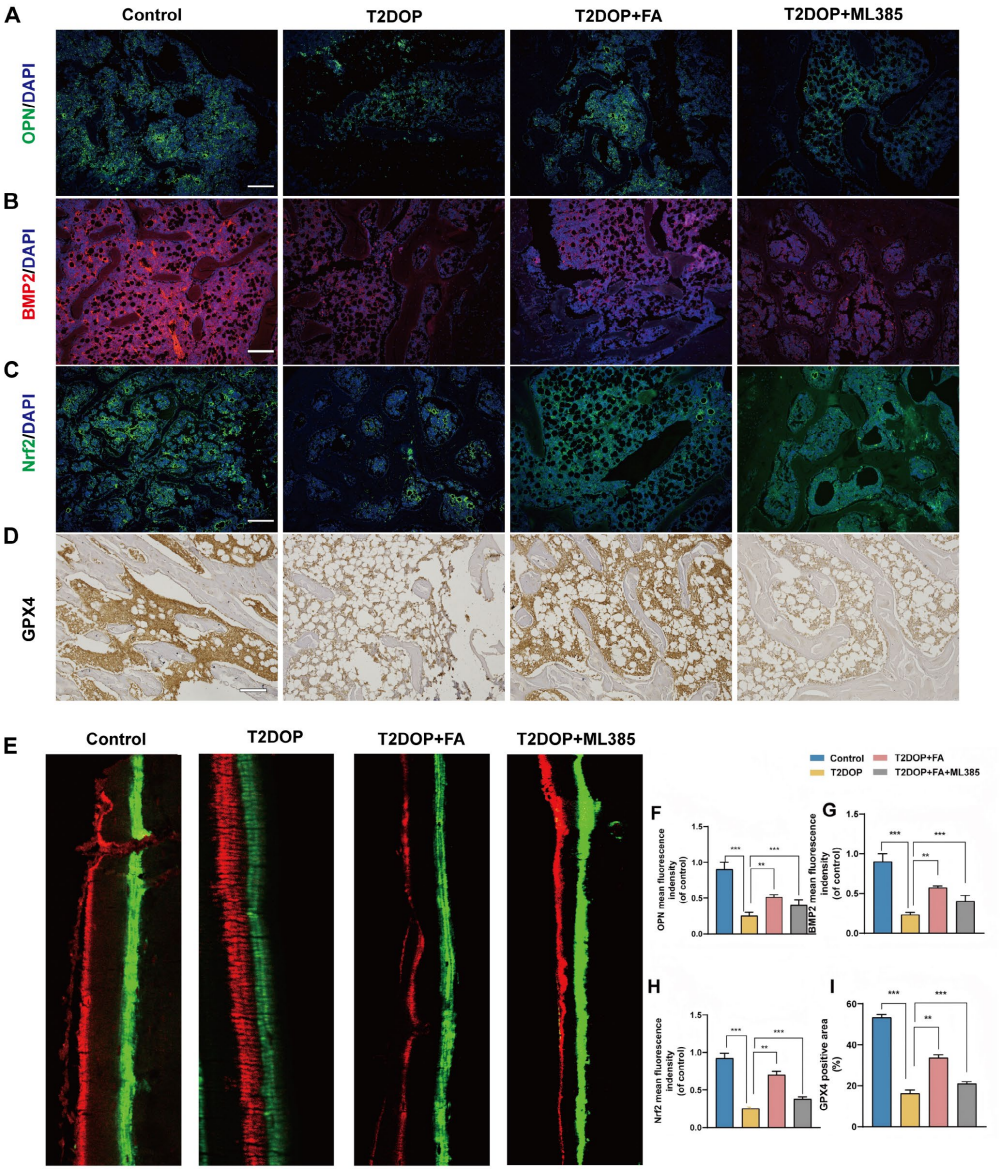
FA treatment increased OPN, BMP2, and Nrf2 expression, promoted bone formation in vivo. (A, F) Immunofluorescence imaging and quantification of OPN in the metaphyseal tissue of the thigh. (B, G) Immunofluorescence imaging and quantitative analysis of BMP2 in thigh metaphyseal tissue. (C, H) Nrf2 immunofluorescence imaging and quantification of metaphyseal thigh tissue. (D, I) Immunohistochemistry staining and quantification analysis of GPX4 protein. (E) Calcein ARS double labeling technique. Images of the mineralized urface of the nondemineralized distal femoral sections. The figures display the mean values ± SEM from five duplicate experiments. ***p < 0.001 compared to the untreated group. GPX4, glutathione peroxidase 4; Nrf2, nuclear factor erythroid 2‐related factor 2. Significance levels: **p < 0.01, ***p < 0.001, ****p < 0.0001.

To assess the correlation between Nrf2 and bone defect repair in rats, we utilized immunofluorescence (IF) to measure the expression levels of relevant proteins in the distal femur. Osteopontin (OPN) and bone morphogenetic protein (BMP2), both of which facilitate bone repair, were selected as indicators, with their fluctuations serving as markers for the extent of repair. Immunofluorescence analysis revealed significantly elevated levels of Nrf2, OPN, and BMP2 in the distal femur of rats treated with FA, compared to those on a high‐fat high‐glucose diet (Figure 8A–C,F–H). Dual labeling using calcein and alizarin red S confirmed previously observed changes in bone structure (Figure 8E). Immunohistochemical analysis ultimately showed that GPX4 levels were higher in the FA‐treated distal femur compared to those in the T2DOP group (Figure 8D,I). The HGHF rat model demonstrated ferroptosis in the distal femur, while FA treatment inhibited this process by activating the Nrf2 pathway.

## 4 Discussion

The global incidence of T2D continues to rise, and its complications, including retinopathy, nephropathy, neuropathy, and vascular diseases, significantly increase the risk of disability and impose substantial economic burdens on patients (Ali et al. 2022). In addition to these well‐known complications, osteoporosis has more recently been recognized as closely associated with the onset of T2D (Hofbauer et al. 2022). Extensive studies have shown that individuals with T2D are at a considerably higher risk of developing osteoporosis compared to healthy individuals without the disease (Chiodini et al. 2020). CT imaging reveals a marked reduction in trabecular bone and a widening of trabecular spaces in T2D patients. These complications are collectively referred to as T2DOP, which often leads to an elevated risk of fractures, posing both health risks and economic challenges (Zoulakis et al. 2024). Consequently, the prevention of T2DOP has become a matter of urgent concern; however, there are currently no definitive therapeutic measures to prevent its onset and progression.

Ferroptosis, a newly identified type of programmed cell death, is associated with multiple organ injuries and degenerative diseases (Li et al. 2020). Ferroptosis is characterized by unique features that differentiate it from other types of programmed cell death. Intracellularly, the buildup of Fe^2+^, elevated ROS production, glutathione depletion, and mitochondrial damage culminate in lipid peroxidation and cell death (Ayala et al. 2014). Additionally, alterations in relevant proteins can serve as indicators of ferroptosis. GPX4, an antioxidant enzyme, plays a critical role in counteracting ferroptosis by scavenging lipid peroxides (Zhang, Liu, et al. 2024). ACSL4 facilitates ferroptosis progression by increasing phospholipid peroxidation. Emerging research has highlighted a significant association between ferroptosis and T2DOP (Liu et al. 2020). Inhibiting ferroptosis to prevent the development and progression of T2DOP is a promising area for future research.

FA, a phenylethanol glycoside derived from *Forsythia suspensa* 's dried fruits, is widely used as a biological agent in scientific studies. It exhibits anti‐inflammatory, antiapoptotic, and neuroprotective properties (Wang et al. 2018; Chen et al. 2019). FA has been confirmed to activate the Nrf2 signaling pathway, exerting antioxidant effects and positioning it as a promising candidate for treating various forms of cell death. Notably, studies have demonstrated that FA can inhibit ferroptosis through Nrf2 activation (Li et al. 2024). Considering ferroptosis as a key element in T2DOP onset and progression, we propose that inhibiting ferroptosis could be a therapeutic approach to reduce T2DOP occurrence and development. To this end, we have initiated an investigation into the potential of FA as a treatment for T2DOP.

**FIGURE 9:**
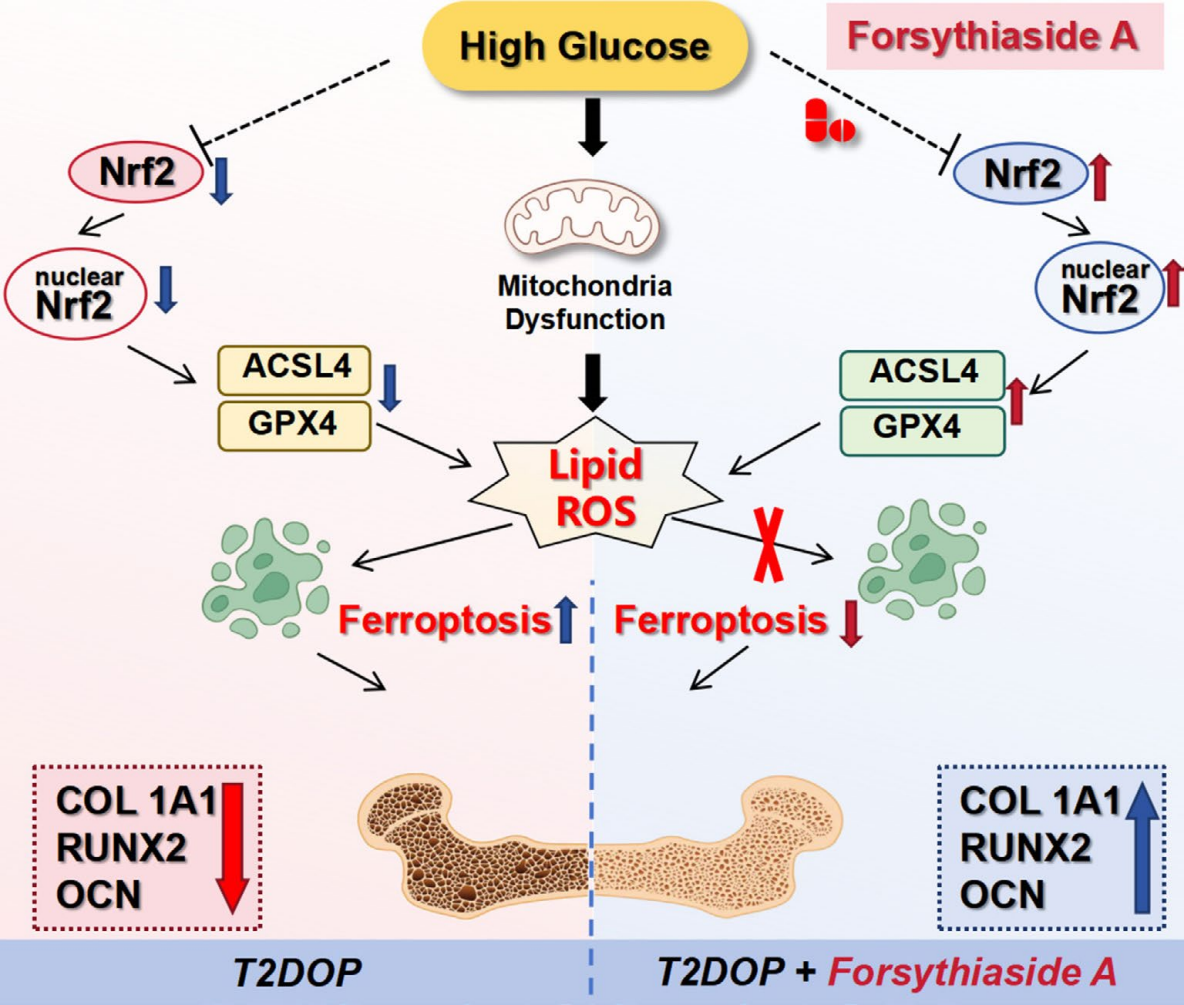
Overview of the pathway involved in FA's effect on HGHF‐induced ferroptosis in BMSCs. FA intervention mitigates HGHF‐induced ferroptosis in bone mesenchymal stem cells (BMSCs) by reducing ROS accumulation and enhancing mitochondrial function. This process enhances T2DOP bone microarchitecture primarily by activating the Nrf2/GPX4 signaling pathway.

We developed both in vitro and in vivo models of T2DOP and administered FA treatment. The findings indicated that FA mitigated ferroptosis and prevented BMSC death by activating the Nrf2 pathway, while also inhibiting BMSC apoptosis, osteogenic differentiation, and mineralization. In vivo, FA successfully counteracted trabecular bone loss, reinstated osteogenic protein expression, and mitigated T2DOP progression. Our data clearly demonstrate that FA has significant potential as a therapeutic agent for T2DOP (Figure 9).

## 5 Conclusion

Our study demonstrates that FA inhibits ferroptosis in BMSCs, likely through the Nrf2/GPX4 axis. Additionally, FA can suppress BMSC apoptosis and restore their osteogenic differentiation capacity. In vivo, FA alleviates the progression of T2DOP in rats. The findings indicate that FA may serve as a promising therapeutic agent for T2DOP treatment.

## Author Contributions

**Yi‐xun Huang:** data curation (equal), formal analysis (equal), visualization (equal), writing – original draft (equal). **Yi‐tian Bu:** conceptualization (equal), data curation (equal), methodology (equal). **Ye‐kai Zhang:** methodology (equal), software (equal). **Yi‐kai Wang:** visualization (equal). **Yang‐fan Guo:** methodology (equal). **Chen Jin:** validation (equal). **Si‐xiang Feng:** software (equal), visualization (equal). **Lai‐fa Kong:** supervision (equal), writing – review and editing (equal). **Wei‐kai Chen:** supervision (equal), writing – review and editing (equal). **Lei Yang:** supervision (equal), writing – review and editing (equal).

## Conflicts of Interest

The authors declare no conflicts of interest.

## Data Availability

The data that support the findings of this study are available on request from the corresponding author.
